# Recurrent stroke risk and cerebral microbleed burden in ischemic stroke and TIA: A meta-analysis

**DOI:** 10.1212/WNL.0000000000003183

**Published:** 2016-10-04

**Authors:** Duncan Wilson, Andreas Charidimou, Gareth Ambler, Zoe V. Fox, Simone Gregoire, Phillip Rayson, Toshio Imaizumi, Felix Fluri, Hiromitsu Naka, Solveig Horstmann, Roland Veltkamp, Peter M. Rothwell, Vincent I.H. Kwa, Vincent Thijs, Yong-Seok Lee, Young Dae Kim, Yining Huang, Ka Sing Wong, Hans Rolf Jäger, David J. Werring

**Affiliations:** From the Stroke Research Centre (D.W., A.C., S.G., P.R., D.J.W.), Department of Brain Repair and Rehabilitation, UCL Institute of Neurology and The National Hospital for Neurology and Neurosurgery; Department of Statistical Science (G.A.) and Biomedical Research Centre (Z.V.F.), UCL, London, UK; Department of Neurosurgery (T.I.), Kushiro City General Hospital, Hokkaido, Japan; Department of Neurology (F.F.), University Hospital Würzburg, Germany; Department of Neurology (H.N.), Suiseikai Kajikawa Hospital, Hiroshima, Japan; Department of Neurology (S.H.), University of Heidelberg, Germany; Department of Stroke Medicine (R.V.), Division of Brain Sciences, Imperial College London; Nuffield Department of Clinical Neurosciences (P.M.R.), John Radcliffe Hospital, University of Oxford, UK; Department of Neurology (V.I.H.K.), Onze Lieve Vrouwe Gasthuis, Amsterdam, the Netherlands; Department of Neurology (V.T.), Austin Health and Melbourne Brain Center, University of Melbourne, Australia; Department of Neurology (Y.-S.L.), Seoul National University Boramae Medical Center; Department of Neurology (Y.D.K.), Yonsei University College of Medicine, Seoul, Korea; Department of Neurology (Y.H.), Peking University First Hospital, Beijing, China; Division of Neurology (K.S.W.), Department of Medicine & Therapeutics, The Chinese University of Hong Kong, Prince of Wales Hospital, Shatin, Hong Kong; and Lysholm Department of Neuroradiology (H.R.J.), National Hospital for Neurology and Neurosurgery, London, UK.

## Abstract

**Objective:**

To determine associations between cerebral microbleed (CMB) burden with recurrent ischemic stroke (IS) and intracerebral hemorrhage (ICH) risk after IS or TIA.

**Methods:**

We identified prospective studies of patients with IS or TIA that investigated CMBs and stroke (ICH and IS) risk during ≥3 months follow-up. Authors provided aggregate summary-level data on stroke outcomes, with CMBs categorized according to burden (single, 2–4, and ≥5 CMBs) and distribution. We calculated absolute event rates and pooled risk ratios (RR) using random-effects meta-analysis.

**Results:**

We included 5,068 patients from 15 studies. There were 115/1,284 (9.6%) recurrent IS events in patients with CMBs vs 212/3,781 (5.6%) in patients without CMBs (pooled RR 1.8 for CMBs vs no CMBs; 95% confidence interval [CI] 1.4–2.5). There were 49/1,142 (4.3%) ICH events in those with CMBs vs 17/2,912 (0.58%) in those without CMBs (pooled RR 6.3 for CMBs vs no CMBs; 95% CI 3.5–11.4). Increasing CMB burden increased the risk of IS (pooled RR [95% CI] 1.8 [1.0–3.1], 2.4 [1.3–4.4], and 2.7 [1.5–4.9] for 1 CMB, 2–4 CMBs, and ≥5 CMBs, respectively) and ICH (pooled RR [95% CI] 4.6 [1.9–10.7], 5.6 [2.4–13.3], and 14.1 [6.9–29.0] for 1 CMB, 2–4 CMBs, and ≥5 CMBs, respectively).

**Conclusions:**

CMBs are associated with increased stroke risk after IS or TIA. With increasing CMB burden (compared to no CMBs), the risk of ICH increases more steeply than that of IS. However, IS absolute event rates remain higher than ICH absolute event rates in all CMB burden categories.

Cerebral microbleeds (CMBs) are radiologically defined small round or ovoid regions of signal loss seen on paramagnetic MRI sequences.^1^ In the limited available pathologic correlation studies, CMBs mostly correspond to hemosiderin-laden macrophages close to vessels affected by small vessel disease.^2–5^ It is thus inferred that CMBs are a marker of direct extravasation of erythrocytes from arterioles and capillaries damaged by bleeding-prone arteriopathies. An arteriopathy associated with systemic arterial hypertension and pathologic changes in small perforating arteries of the deep gray and white matter causes CMBs in deep (basal ganglia) as well as lobar regions. In Western (Caucasian) people with intracerebral hemorrhage (ICH), CMBs in a strictly lobar distribution are highly specific for cerebral amyloid angiopathy (CAA), which causes progressive deposition of β-amyloid in small cortical and leptomeningeal arterial walls,^6^ though this pattern may not be so specific in Eastern (Asian) people^7^ and in those without ICH.^8^

Multiple prospective studies in ischemic stroke (IS) cohorts have shown that CMBs are associated with subsequent ICH risk.^9,10^ However, CMBs are also associated with increased subsequent IS risk.^11–14^ Indeed, suggested ischemic mechanisms for CMBs include ischemia-mediated iron store release by oligodendrocytes,^15^ phagocytosis of red cell microemboli into the perivascular space (termed angiophagy),^16^ or hemorrhagic transformation of small microinfarcts.^17^ Indeed, in a recent community study, after adjusting for cardiovascular risk factors, CMBs were found to be associated with lacunes and white matter volume progression.^18^ Few data are available on how CMB burden affects the balance of ICH and IS risk in different populations. In CAA cohorts, an increasing number of CMBs is associated with an increased risk of ICH, suggesting a relationship between CMB number and the severity of bleeding-prone arteriopathy.^19^ Whether an increasing number of CMBs is also associated with an increased risk of ICH in IS and TIA cohorts remains uncertain. If increasing CMB burden shifts the balance of risk toward ICH rather than IS, this could have major clinical relevance for antithrombotic risk-benefit decisions after IS and TIA. Our previous meta-analysis of 10 prospective studies including 3,067 patients with IS or TIA found that CMB presence is associated with a higher risk of ICH than IS^20^ (the odds ratio was 8.53 for ICH and 1.55 for IS), but was not able to address the key clinical question of how the number (burden) of CMBs influences ICH and IS risk.

We therefore performed a pooled analysis of aggregate summary data, including CMB burden and distribution, to investigate the risk of subsequent IS and ICH in individuals who have had an IS or TIA. We tested the following hypotheses: (1) CMB presence is associated with an increased risk of stroke (ICH > IS); and (2) as CMB burden increases (due to a more severe bleeding-prone arteriopathy), the risk of ICH increases more steeply than the risk of IS.

## METHODS

We searched Medline and Embase from 1996 (the year CMBs were first reported) through to April 2015. Our search strategy was as follows:

1. “Cerebral microbleed*” or CMB or “cerebral microh?emorr*” or “brain microbleed*” or “brain microh?emorr*”
2. Stroke or “isch?emic stroke” or TIA or “intrac* adj2 h?emorrhag*” or ICH
3. 1 and 2

We included published and unpublished studies fulfilling the following criteria: (1) performed paramagnetic-sensitive MRI sequences to detect CMBs at baseline; (2) assessed CMBs at baseline and associations with IS or ICH as primary or secondary outcomes; (3) had a prospective study design with at least 3 months of follow-up; and (4) fulfilled at least 4 of 6 predefined quality indicators. We excluded cross-sectional studies and case series. Two clinical research fellows (A.C. and D.W.) reviewed each study for eligibility.

### Data extraction.

We contacted all authors to provide data on study population, size, patient-year follow-up, and antithrombotic treatment. We obtained data on outcome events of symptomatic IS and ICH, with baseline CMB number categories as follows: CMB present, 1 CMB, >1 CMB, 2–4 CMBs, 5–10 CMBs, >10 CMBs, strictly deep CMBs, strictly lobar CMBs, and mixed distribution CMBs. We extracted all demographic, imaging, and follow-up outcome data from each study.

### Quality assessment and reducing the risk of bias.

All included studies were critically appraised against a checklist of 6 key quality indicators (table e-1 at Neurology.org), with reference to the Strengthening the Reporting of Observational Studies in Epidemiology statement and the Preferred Reporting Items for Systematic Reviews and Meta-Analyses guidelines. The quality criteria included assessment for bias, and all studies had a quality score of ≥4/6.

### Statistical methods.

We first performed separate random effect meta-analyses to derive summary estimates of the pooled risk ratios of ICH and IS for each CMB category vs the reference category of “no CMBs.” Due to the small number of events in some studies, the categories 5–10 and >10 were combined, in line with previous studies of CMB burden and prognosis, which demonstrated their prognostic relevance for future ICH risk.^19^ Logistic regression was then used to estimate the increased risk (odds) of IS/ICH for each additional CMB. First, CMB categories were converted to a continuous scale by assuming that patients in the CMB groups 0, 1, 2–4, 5–10, and >10 had, on average, 0, 1, 3, 7.5, and 12.5 CMBs, respectively. Then, for each study, a logistic regression model was fitted relating the (log) odds of IS or ICH to the (estimated) number of CMBs. These (log) odds ratios were then pooled using random effects meta-analysis.

We calculated the *I*^2^ statistic to investigate heterogeneity. Funnel plots (Begg and Mazumdar) were generated to investigate publication bias. Finally, where necessary, we undertook meta-regression of confounding covariates of biological plausibility or with differences between the studies (average follow-up, age, hypertension prevalence, demographics, antithrombotic use). All analyses were performed using STATA 12.0 (StataCorp LP, College Station, TX).

### Standard protocol approvals, registrations, and patient consents.

Individual studies and data transfer protocols were approved by local ethics committees. No additional ethical approval was required for this meta-analysis.

## RESULTS

**Figure 1:**
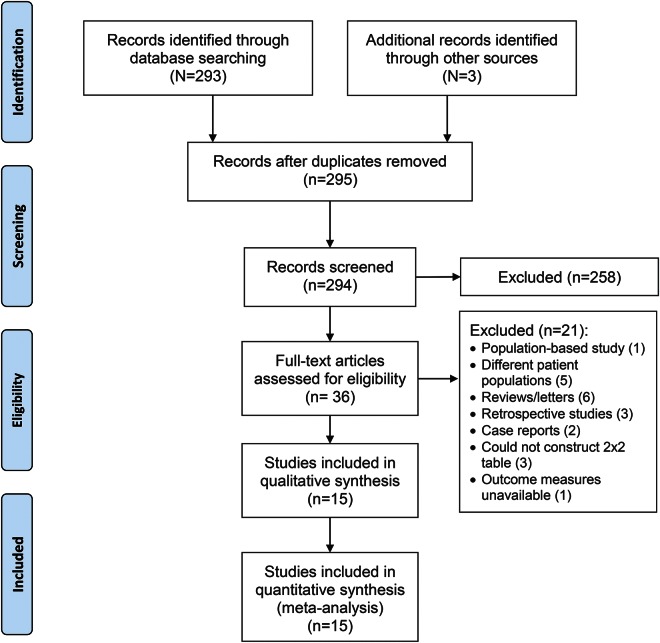
Preferred Reporting Items for Systematic Reviews and Meta-Analyses flow chart of study selection

Fifteen studies met our inclusion criteria (12 published and 3 unpublished) including 5,068 patients^9,11–14,21–27^ (figure 1). The patient and data characteristics of each study are shown in tables e-2 and e-3. Twelve of the 15 studies provided data on CMBs at the time of the initial IS/TIA fully stratified into number categories and location. Eight studies involving 3,111 patients were from predominantly Eastern (Asian) cohorts; the remainder (1,957 patients) were predominantly Western (Caucasian). Two studies included strictly TIA patients, 4 included those with TIA or IS, and 9 included strictly IS patients. The number of patients with CMBs was 1,284, giving an overall pooled prevalence of 25.3%. Median follow-up was 18 months (interquartile range 11–30). Overall, 79% of patients were prescribed antiplatelet agents; only 15% of patients were prescribed anticoagulants (mainly from one study with a high proportion of patients on anticoagulation [87%]).^24^ CMB presence was more prevalent, with higher burden, in the Eastern cohorts compared to Western cohorts (table e-3).

### CMBs and IS risk.

The total recurrent IS rate was 327/5,068 (6.5%). The IS event rate in those with CMBs was 9% (115/1,284) vs 5.6% (212/3,781) for those without CMBs; thus, CMBs are associated with an absolute risk increase of 3.4% for IS. The absolute risk increase for IS for CMBs vs no CMBs increases as the CMB burden increases (1.8% for 1 CMB, 4.8% for 2–4 CMBs, and 5.1% for ≥5 CMBs [table 1]).

**Table 1:**
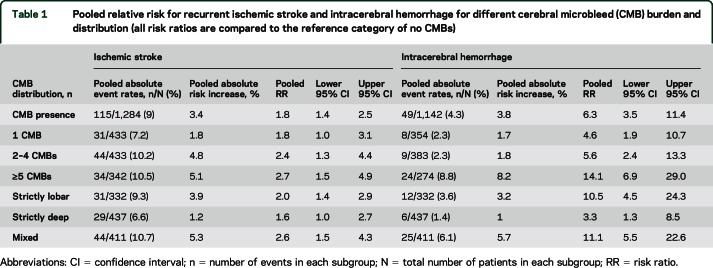
Pooled relative risk for recurrent ischemic stroke and intracerebral hemorrhage for different cerebral microbleed (CMB) burden and distribution (all risk ratios are compared to the reference category of no CMBs)

**Figure 2:**
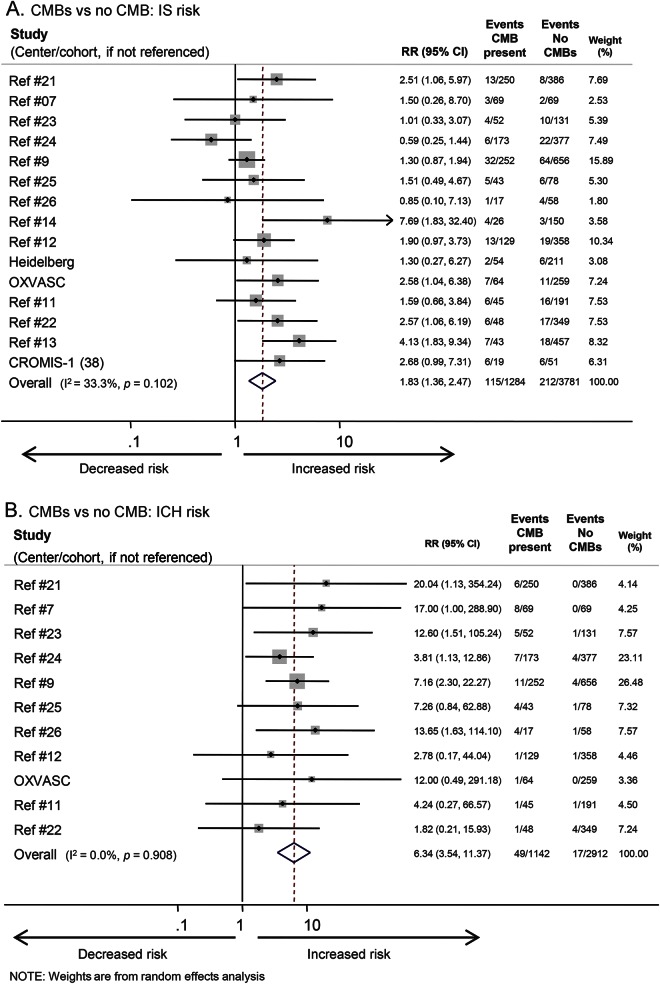
Forest plot of the risk of ischemic stroke (IS) and intracerebral hemorrhage (ICH) for presence of any cerebral microbleeds (CMBs) vs no CMBs CI = confidence interval; RR = risk ratio.

**Figure 3:**
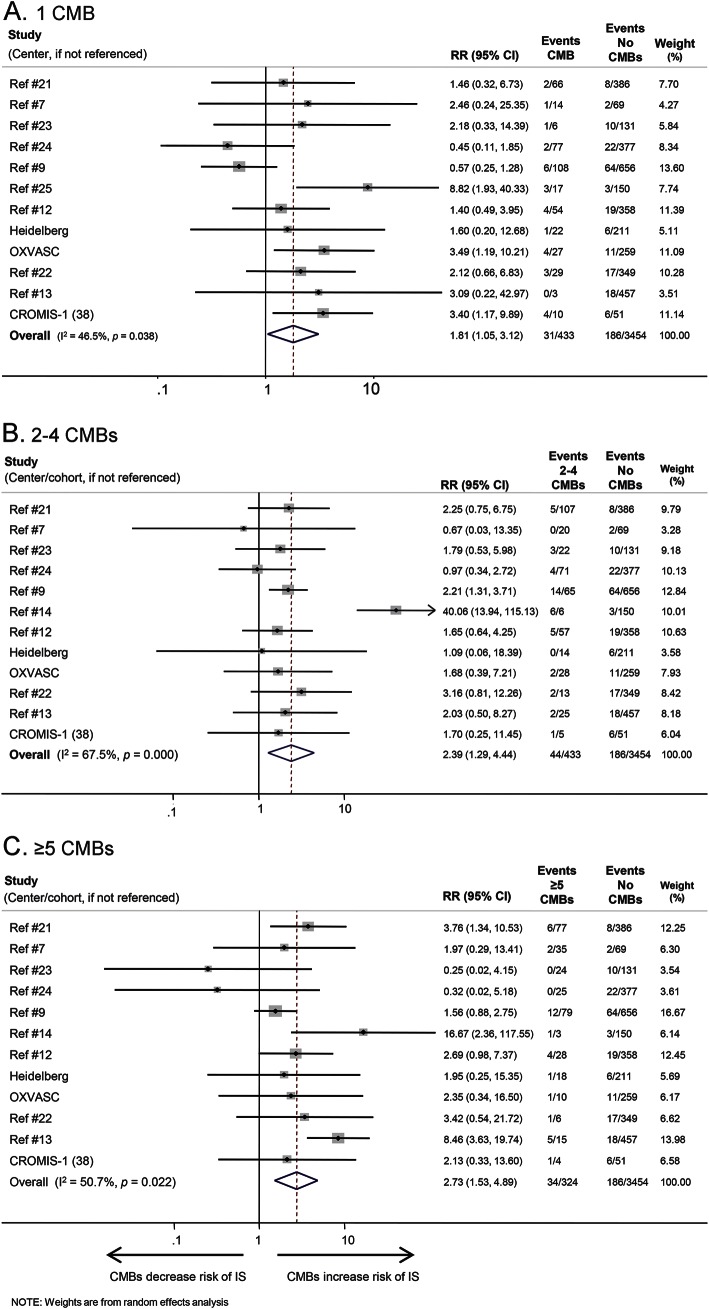
Forest plot of the risk of ischemic stroke (IS) for different burdens of cerebral microbleeds (CMBs) vs no CMBs CI = confidence interval; RR = risk ratio.

The risk ratios for different CMB burden and distribution categories on IS are also shown in table 1. The presence of CMBs (vs no CMBs) was associated with a pooled risk ratio of recurrent IS of 1.8 (95% confidence interval [CI] 1.4–2.5) (figure 2). Funnel plots revealed no evidence of publication bias (Egger test *p* = 0.4). The presence of a single CMB (vs no CMBs) had a pooled risk ratio for IS of 1.8 (95% CI 1.0–3.1). The pooled risk estimates for IS suggest an increasing trend toward higher IS risk with increasing CMB burden (table 1 and figure 3). Logistic regression was used to estimate the increase in risk for each additional CMB; this showed an odds ratio of 1.10 (95% CI 1.06–1.14) per CMB increase (figure e-1). The risk estimates for IS for each distribution category of CMBs (vs no CMBs) ranged from 1.6 (95% CI 1.0–2.7) for strictly deep CMBs to 2.6 (95% CI 1.5–4.3) for mixed CMBs, with overlapping confidence intervals for all groups. Because we noted statistical heterogeneity among the cohorts for IS risk (*I*^2^ 33%, 47%, 68%, and 51%, respectively, for CMB presence, 1 CMB, 2–4 CMB, and ≥5 CMB, respectively), each potential confounder (ethnicity, average follow-up, age, hypertension prevalence, and antithrombotic use) was investigated separately using meta-regression (table e-4, figure e-2). We found only weak evidence for a confounding effect of hypertension; as expected, the relative risk associated with baseline CMBs on future IS risk (effect size) is attenuated in studies with a higher prevalence of hypertension.

### CMBs and ICH risk.

The total ICH rate was 66/5,068 (1.3%). The ICH event rate in those with CMBs was 4.3% (49/1,142) vs 0.5% (17/2,912) for those without CMBs; thus, CMBs confer an absolute risk increase of 3.8% for ICH. The absolute risk increase for ICH for CMBs vs no CMBs increases as the CMB burden increases (1.7% for 1 CMB, 1.8% for 2–4 CMBs, and 8.2% for ≥5 CMBs [table 1]).

**Figure 4:**
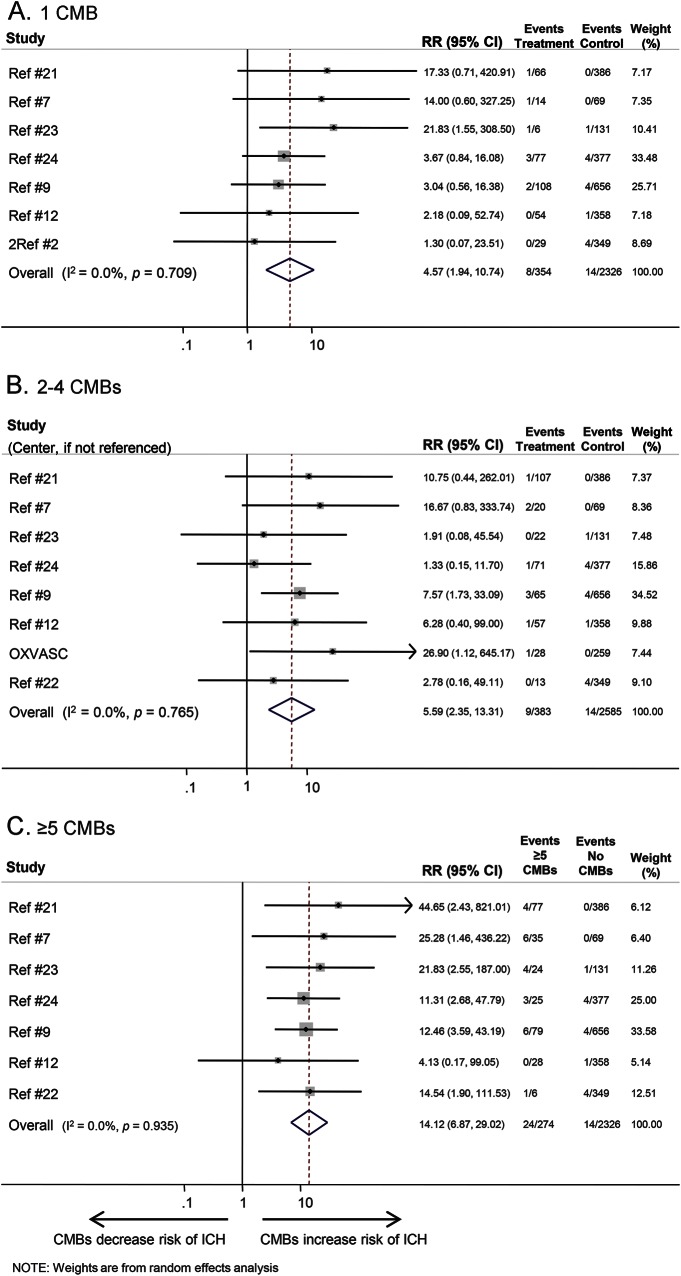
Forest plot of the risk of intracerebral hemorrhage (ICH) for different burdens of cerebral microbleeds (CMBs) vs no CMBs CI = confidence interval; RR = risk ratio.

The risk ratios for different CMB burden and distribution categories on ICH are also shown in table 1.The presence of CMBs (vs no CMBs) was associated with a pooled risk ratio of 6.3 for subsequent ICH (95% CI 3.5–11.4) (figure 2). Four studies were excluded, as they did not report any ICH outcomes. Increasing CMB burden was associated with an increased risk of ICH (pooled risk ratio 4.6 [95% CI 1.9–10.7], 5.6 [95% CI 2.4–13.3], and 14.1 [95% CI 6.9–29.0] for 1 CMB, 2–4 CMBs, and ≥5 CMBs compared to no CMBs, respectively) (table 1 and figure 4). Logistic regression showed an odds ratio of 1.29 (95% CI 1.21–1.37) for ICH per additional CMB (figure e-1). Of the CMB anatomical distribution categories (strictly lobar, mixed, or strictly deep), strictly lobar CMBs were associated with the highest risk of subsequent ICH vs no CMBs (pooled risk ratio 10.5 [95% CI 4.5–24.3]; table 1). There was no publication bias within studies (Egger test *p* = 0.98). Meta-regression was not undertaken because heterogeneity was not detected (*I*^2^ was 0%) for ICH outcomes.

## DISCUSSION

Our meta-analysis of 15 prospective studies, including more than 5,000 patients presenting with IS or TIA, found that the presence of any CMBs is associated with an approximate doubling of the risk of IS, but with an approximately 6-fold increase in the risk of ICH, in keeping with 2 previous smaller meta-analyses.^20,28^ Our meta-analysis also builds on these previous studies and adds new knowledge on ICH risk: first, we were able to increase our statistical power by including more ICH outcomes^20^; second, by pooling aggregate data, we investigated how increasing CMB burden affects the balance between future IS and ICH (including both relative and absolute risks); and third, we partially adjusted for confounding factors through meta-regression. Our most important new finding is that with increasing CMB burden, the risk of ICH increases more steeply than that of IS. In patients with ≥5 CMBs, the risk of ICH was substantially higher than that of IS (risk ratio for ICH 14.1 [95% CI 6.7–29.0] vs risk ratio for IS 2.73 [95% CI 1.5–4.9]). In a complementary logistic regression analysis, we showed that each additional CMB is associated with an increased odds of 1.3 (95% CI 1.2–1.4) for ICH and 1.1 (95% CI 1.1–1.1) for IS, supporting a steeper increase in ICH than IS risk with higher CMB burden. However, the absolute event rate of recurrent IS was consistently higher than the absolute event rate of ICH in patients with CMBs and within all CMB categories, including those with ≥5 CMBs.

A large number of CMBs (e.g., ≥5 CMB) might help identify patients at substantially higher risk of ICH than of IS. Indeed, for clinicians, the key question is what burden of CMBs could tip the balance of risk towards ICH sufficiently to affect clinical decisions; for example, antithrombotic drug use. Antiplatelet agents only modestly reduce the absolute risk of IS in secondary prevention (0.5%–2.5%).^29^ Our data show the absolute risk of ICH increases substantially more than the absolute risk of IS as CMB burden increases; the effect is most evident with ≥5 CMBs, which is associated with an 8.2% absolute risk increase for ICH vs a 5.1% absolute risk increase for IS. This raises the possibility that antiplatelet drug risk-benefit assessment may favor avoiding their use in those with numerous CMBs (e.g., ≥5 CMBs). This could affect a substantial proportion of IS and TIA patients; of patients with CMBs, the prevalence of those with ≥5 CMBs ranged from 12% to 51% in studies included in this analysis (table e-3) and varied by ethnicity (mean 17% for Western cohorts and 35% for Eastern cohorts).

A previous meta-analysis suggested that ethnicity may be an important determinant of the balance of ICH and IS risks associated with CMBs; an increased risk for ICH risk was only statistically significant in Eastern cohorts, while IS risk was only significant in Western cohorts.^20^ In the present study, we included more patients, and using meta-regression found that ethnicity does not confound the association between CMB burden and IS or ICH risks. Thus, based on the current study, CMB burden appears to be a greater predictor of IS and ICH risk than ethnicity.

Our study has a number of strengths, including a large sample size from multiple cohorts from different countries. We only included those of high quality using systematic quality indicator assessment. We included data on CMB burden and distribution, and adjusted for confounding factors through meta-regression. Our study thus provides the best currently available evidence on how CMBs affect IS and ICH risk after IS or TIA.

Our study also has limitations. Because we included aggregate summary-level data (rather than individual patient data), we could not explore the effect of CMB distribution free from the confounding effect of CMB burden. The mixed CMB category has the highest risk of stroke, but by definition includes only patients with multiple CMBs; by contrast, the strictly lobar and strictly deep CMB categories could include patients with a single CMB. Specifically, we could not fully investigate the independent risk associated with strictly lobar CMBs, critical to the diagnosis of CAA^4^ with high recurrent ICH risk.^19,30^ Although we undertook meta-regression, this can only partially account for confounding and is unlikely to fully account for variables such as age and hypertension. Our logistic regression assumes an average CMB count within each category (difficult to estimate in the open-ended ≥10 CMB category) and that the log odds of ICH/IS increase linearly with CMB burden. However, consistent findings from 2 complementary statistical analyses strongly support the hypothesis that increasing CMB burden increases the risk of ICH more than that of IS. Further limitations include the variable study sample size and follow-up, which may bias our results, especially regarding ICH, a rare outcome with wide CIs around risk estimates (which overlap for the different CMB burden categories). A time to event analysis may have been a more appropriate statistical method given the varying follow-up, but this was not possible with the data available. Imaging protocols and analysis were similar but not completely uniform (table e-2); field strength,^31^ echo time,^32^ and optimized paramagnetic sequences^33^ can all influence CMB detection. However, most studies were performed at 1.5T with echo times within a narrow range, making this unlikely to affect our conclusions. Nevertheless, our results are only generalizable to patients scanned on 1.5T MRI using gradient recalled echo and may not be applicable to susceptibility-weighted imaging (SWI) or MRI with a higher field strength. SWI increases the number of CMBs detected^33,34^ compared to T2*-weighted images; CMB burden categories may thus have to be revised for SWI. Finally, studies used different methods for CMB rating; standardized rating instruments^1,35,36^ may improve the reliability of defining CMB categories, particularly that of a single CMB.

Although our study provides important new information, to fully determine how CMBs might influence antithrombotic decisions, the interaction between CMBs and antiplatelet agents and anticoagulants needs to be further addressed in large prospective studies. Although the prevalence of antithrombotic and anticoagulant use did not show an association with either ICH or IS outcome in our meta-regression, the large majority of patients we included were treated with antiplatelet agents. Very few patients included in our meta-analysis were on anticoagulation; more data are therefore needed on this group, who may be at highest ICH risk. Ongoing prospective observational studies addressing this question include ucl.ac.uk/cromis-2^37^ and clinicaltrials.gov/ct2/show/NCT02238470. Further pooled analyses of individual patient data from these and other observational studies—and, ultimately, randomized controlled trials based on CMB burden—are needed to fully assess the interaction between CMBs and antithrombotic drugs (both antiplatelet agents and anticoagulants) after IS and TIA. Nevertheless, we have shown that with increasing CMB burden, the risk of ICH increases more steeply than that of IS in a cohort of IS and TIA patients largely treated with antiplatelet medication. A high CMB burden (e.g., ≥5 CMBs) may identify patients at similar or greater risk of ICH than IS, with implications for antithrombotic treatment and future randomized controlled trials.

## Supplementary Material

Data Supplement

## GLOSSARY

CAA: cerebral amyloid angiopathy
CI: confidence interval
CMB: cerebral microbleed
ICH: intracerebral hemorrhage
IS: ischemic stroke
SWI: susceptibility-weighted imaging
