# Supplementary material for: Recurrent stroke risk and cerebral microbleed burden in ischemic stroke and TIA: A meta-analysis
Source: Neurology. 2016 Oct 4;87(14):1501–10. doi: 10.1212/WNL.0000000000003183 (PMC5075978; doi:10.1212/WNL.0000000000003183)
Supplement: Data Supplement [file supp_87_14_1501__index.html]

Recurrent stroke risk and cerebral microbleed burden in ischemic stroke and TIA — Data Supplement 

# Recurrent stroke risk and cerebral microbleed burden in ischemic stroke and TIA

## Data Supplement

**Neurology® data supplements are not copyedited before publication. Published editorials and translations have been copyedited.  
 © 2016 American Academy of Neurology.  
  
 Files in this Data Supplement:**

- Figure e-1 - PDF
- Figure e-2 - PDF
- Table e-1 - PDF
- Table e-2 - PDF
- Table e-3 - PDF
- Table e-4 - PDF
