# Supplementary material for: Recurrent stroke risk and cerebral microbleed burden in ischemic stroke and TIA: A meta-analysis
Source: Neurology. 2016 Oct 4;87(14):1501–10. doi: 10.1212/WNL.0000000000003183 (PMC5075978; doi:10.1212/WNL.0000000000003183)
Supplement: Data Supplement [file supp_WNL.0000000000003183_figure_e-1.pdf]

Online supplement Figure e-1. Forest plot showing logistic regression meta-analysis of CMBs vs. no CMBs for IS and ICH outcomes

Logistic regression meta-analysis for IS

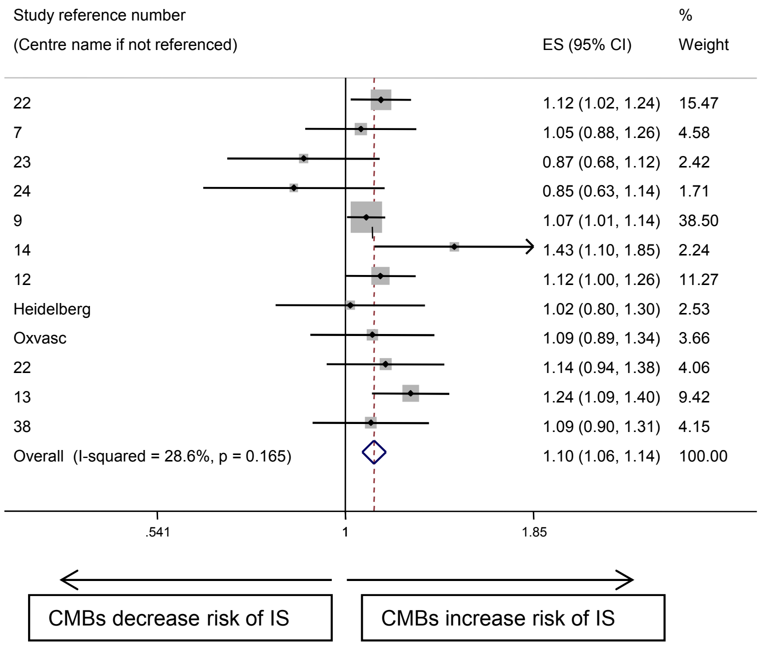

Logistic regression meta-analysis for ICH

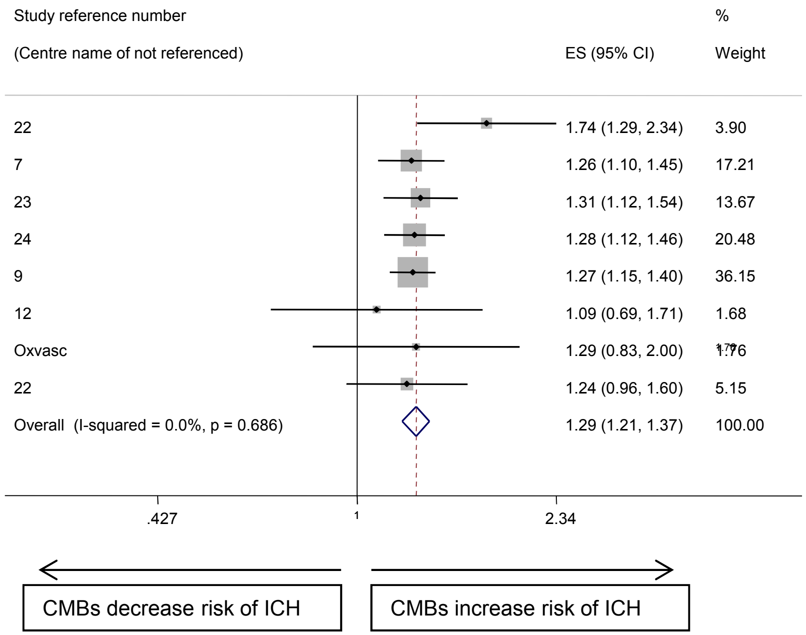

Legend: CMB- cerebral microbleed, IS –ischemic stroke, ICH Intracerebral hemorrhage
