# Supplementary material for: Recurrent stroke risk and cerebral microbleed burden in ischemic stroke and TIA: A meta-analysis
Source: Neurology. 2016 Oct 4;87(14):1501–10. doi: 10.1212/WNL.0000000000003183 (PMC5075978; doi:10.1212/WNL.0000000000003183)
Supplement: Data Supplement [file supp_WNL.0000000000003183_figure_e-2.pdf]

# Online supplement Figure e-2. Meta-regression plots showing the relationship between intra-study variables and the effect of CMBs on the relative risk of future ischemic stroke

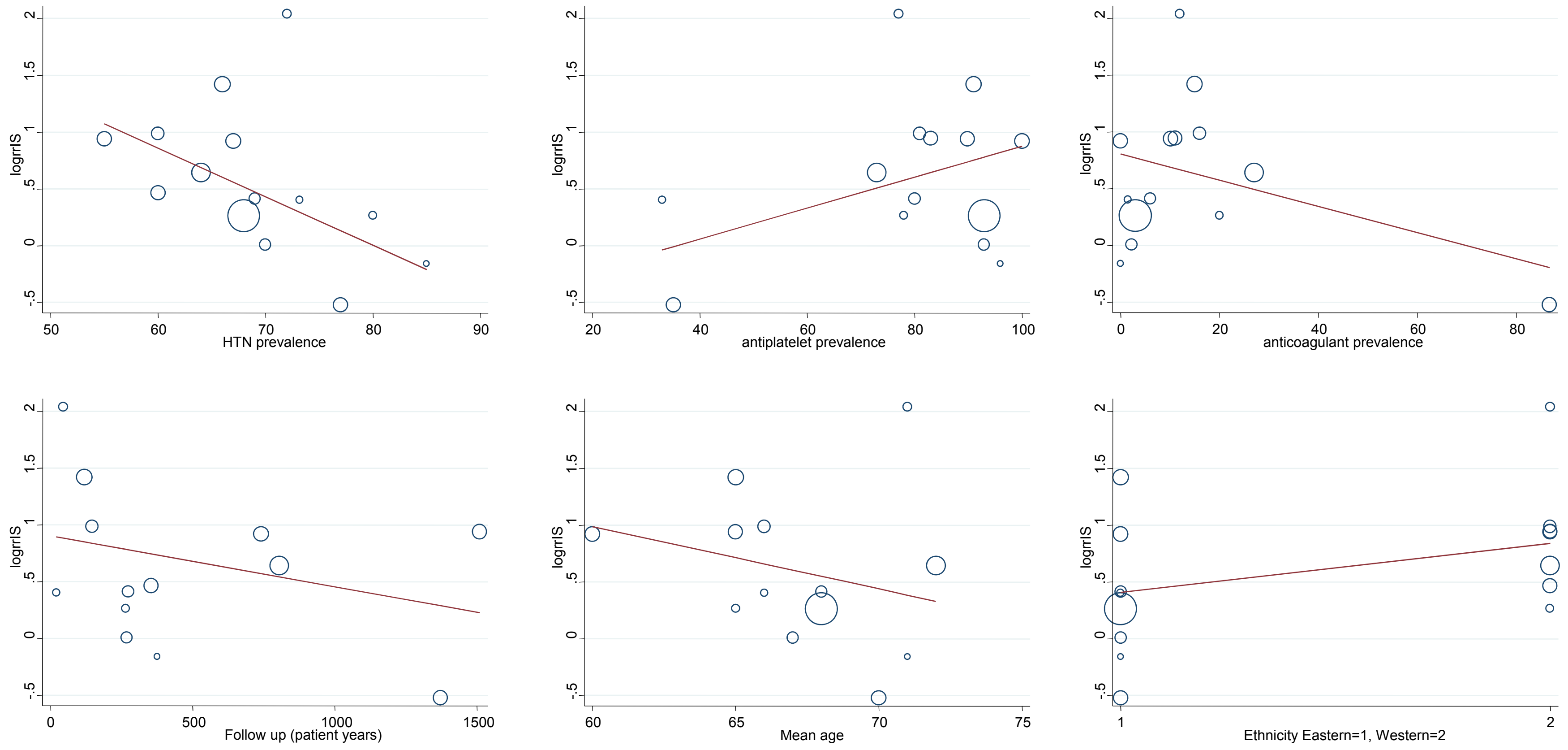


HTN -hypertension
