# Supplementary material for: Recurrent stroke risk and cerebral microbleed burden in ischemic stroke and TIA: A meta-analysis
Source: Neurology. 2016 Oct 4;87(14):1501–10. doi: 10.1212/WNL.0000000000003183 (PMC5075978; doi:10.1212/WNL.0000000000003183)
Supplement: Data Supplement [file supp_WNL.0000000000003183_Table_e-1.pdf]

Online supplement table e-1: Quality indicators for the included studies

Study (primary author or name) and Ref	Clearly defined population	Standardised MRI parameters	CMB clearly defined	Standardised rating scale	Standardised definition of outcome	Completion of follow up (>90%)	Number of quality indicators
Boulanger (11)	+	+	+	?	+	+	5/6
CROMIS-1* (e1)	+	-	+	+	+	-	4/6
Fan (25)	+	+	+	?	+	+	5/6
Fluri (14)	+	+	+	+	+	+	6/6
Heidelberg*	+	+	+	+	+	+	6/6
Huang (22)	+	-	+	?	+	+	4/6
Imaizumi (07)	+	+	+	?	+	+	5/6
Kwa (22)	+	+	+	+	+	+	6/6
Lim (13)	+	+	+	+	+	+	6/6

Mok (26)	+	+	+	?	+	-	4/6
Naka (23)	+	+	+	?	+	+	5/6
OXVASC*	+	+	+	+	+	+	6/6
Song (24)	+	+	+	+	+	+	6/6
Soo (9)	+	+	+	?	+	+	5/6
Thijs (12)	+	-	+	+	+	+	5/6

Legend * Denotes unpublished study. + = present; - = absent; ? = unavailable. CMB – cerebral microbleed

e-Reference

e1. CROMIS 1 [online]. Available at: http://public.ukcrn.org.uk/search/StudyDetail.aspx?StudyID=4152 [accessed 21/12/2015].
