# Supplementary material for: Recurrent stroke risk and cerebral microbleed burden in ischemic stroke and TIA: A meta-analysis
Source: Neurology. 2016 Oct 4;87(14):1501–10. doi: 10.1212/WNL.0000000000003183 (PMC5075978; doi:10.1212/WNL.0000000000003183)
Supplement: Data Supplement [file supp_WNL.0000000000003183_Table_e-2.pdf]

Online supplement table e-2: Patient characteristics of studies included

Study (primary author or name) Ref	Number of patients	Average follow up (months)	Ethnicity	Cohort	Age (mean)	HTN (%)	Gender (% Male)	Antiplatelets (%)	Anticoagulants (%)	Field strength (Tesla)	Echo time (ms)	T2*/SWI
Boulanger (11)	236	18	Western	IS/TIA	NA	60	55	NA	NA	3	20	T2*
CROMIS-1 (e1)*	68	24	Western	IS/TIA	66	60	66	81	16	1.5	Variable	T2*
Fan (25)	121	27	Eastern	IS	68	69	68	80	6	1.5	30	T2*
Fluri (14)	176	3	Western	TIA	71	72	61	77	12	1.5	15	T2*
Heidelberg*	265	12	Western	IS	65	80	67	78	20	3	19.7	SWI
Huang (21)	636	14	Eastern	IS	60	67	68	100	0	1.5	NA	T2*
Imaizumi (07)	138	22	Eastern	IS	66	73.2	66	33	2	1.5	26	T2*
Kwa (22)	397	46	Western	IS	65	55	59	90	10	1.5	27.6	T2*
Lim (13)	500	3	Eastern	TIA	65	66	58	91	15	NA	15-25	T2*

Mok (26)	75	60	Eastern	IS	71	85	52	96	0	1.5	NA	T2*
Naka (23)	183	18	Eastern	IS	67	70	63	93	2	1	26	T2*
OXVASC*	323	35	Western	IS/TIA	72	63	75	83	11	1.5	14	T2*
Song (24)	550	30	Eastern	IS	70	77	59	35	87	3	16	T2*
Soo (9)	908	11	Eastern	IS	68	68	58	93	3	1.5	30	T2*
Thijs (12)	487	20	Western	IS/TIA	72	64	61	73	27	Variable	Variable	T2*

Legend: IS-* denoted unpublished study, Ischemic stroke; TIA- transient ischemic attack, HTN –hypertension SWI -susceptibility weighted imaging

e-Reference

e1. CROMIS 1 [online]. Available at: http://public.ukcrn.org.uk/search/StudyDetail.aspx?StudyID=4152 [accessed 21/12/2015].
