# Supplementary material for: Recurrent stroke risk and cerebral microbleed burden in ischemic stroke and TIA: A meta-analysis
Source: Neurology. 2016 Oct 4;87(14):1501–10. doi: 10.1212/WNL.0000000000003183 (PMC5075978; doi:10.1212/WNL.0000000000003183)
Supplement: Data Supplement [file supp_WNL.0000000000003183_Table_e-3.pdf]

Online supplement Table e-3: Individual study cerebral microbleed (CMB) prevalence and distribution

		Prevalence for different anatomical distributions and CMB counts in patients with CMBs		
**Study (primary author or name) Ref**	**CMB prevalence n/N (%)**	**Strictly Deep CMB prevalence n/N (%)**	**Strictly lobar CMB prevalence n/N (%)**	**≥ 5 CMBs prevalence n/N (%)**
Boulanger (11)	45/236 (19)	NA	NA	NA
CROMIS 1 (e1)*	19/73 (25)	6/19 (32)	6/19 (32)	4/19 (21)
Fan (25)	43/121(36)	NA	NA	NA
Fluri (14)	26/176 (15)	6/26 (23)	15/26 (58)	3/26 (12)
Heidelberg*	54/265 (20)	10/54 (19)	21/54 (38)	18/54 (33)
Huang (21)	250/636 (39)	101/250 (40)	51/250 (20)	77/250 (31)
Imaizumi (07)	69/138 (50)	31/69 (45)	4/69 (6)	35/69 (51)
Kwa (22)	48/397 (12)	19/48 (40)	19/48 (40)	6/48 (13)
Lim (13)	43/500 (9)	23/42 (55)	7/43 (16)	15/43 (35)

Mok (26)	17/75 (23)	NA	NA	NA
Naka (23)	52/183 (28)	26/52 (50)	5/52 (10)	24/52 (46)
OXVASC*	64/323 (20)	13/64 (20)	34/64 (53)	10/64 (16)
Song (24)	173/550 (31)	77/173 (45)	44/173 (25)	25/173 (14)
Soo (9)	252/908 (28)	100/252 (40)	67/252 (27)	79/252 (31)
Thijs (12)	129/487 (26)	25/129 (19)	59/129 (46)	28/129 (22)

Legend: * denotes unpublished study, CMB –Cerebral microbleed

e-Reference

e1. CROMIS 1 [online]. Available at: http://public.ukcrn.org.uk/search/StudyDetail.aspx?StudyID=4152 [accessed 21/12/2015].
