# Supplementary material for: Recurrent stroke risk and cerebral microbleed burden in ischemic stroke and TIA: A meta-analysis
Source: Neurology. 2016 Oct 4;87(14):1501–10. doi: 10.1212/WNL.0000000000003183 (PMC5075978; doi:10.1212/WNL.0000000000003183)
Supplement: Data Supplement [file supp_WNL.0000000000003183_Table_e-4.pdf]

**Online supplement Table e-4: Univariable meta-regression showing the effect on heterogeneity of each variable for the association of recurrent ischemic stroke risk and CMB presence**

| Variable | Reduction in $I^2$ % | p value of regression coefficient in meta-regression |
|---|---|---|
| Ethnicity (East compared to West) | 8.3 | 0.17 |
| Patient year follow up | 4.9 | 0.23 |
| Age (year) | 2.3 | 0.33 |
| Hypertension % | 14.8 | 0.08 |
| Anticoagulant use % | 4.3 | 0.13 |
| Antiplatelet use % | 3.4 | 0.15 |

HTN –hypertension, CMB-cerebral microbleed
